# Cyanophycin Mediates the Accumulation and Storage of Fixed Carbon in Non-Heterocystous Filamentous Cyanobacteria from Coniform Mats

**DOI:** 10.1371/journal.pone.0088142

**Published:** 2014-02-07

**Authors:** Biqing Liang, Ting-Di Wu, Hao-Jhe Sun, Hojatollah Vali, Jean-Luc Guerquin-Kern, Chung-Ho Wang, Tanja Bosak

**Affiliations:** 1 Institute of Earth Sciences, Academia Sinica, Nangang, Taipei, Taiwan, ROC; 2 Department of Earth, Atmospheric and Planetary Sciences, Massachusetts Institute of Technology, Cambridge, Massachusetts, United States of America; 3 Department of Earth Sciences, National Cheng Kung University, Tainan 701, Taiwan, ROC; 4 INSERM, U759, Orsay, France; 5 Institut Curie, Laboratoire de Microscopie Ionique, Orsay, France; 6 Department of Life Sciences, National Central University, Jhongli City, Taiwan, ROC; 7 Facility for Electron Microscopy Research, McGill University, Montreal, Canada; 8 Department of Anatomy and Cell Biology, McGill University, Montreal, Canada; University of New South Wales, Australia

## Abstract

Thin, filamentous, non-heterocystous, benthic cyanobacteria (Subsection III) from some marine, lacustrine and thermal environments aggregate into macroscopic cones and conical stromatolites. We investigate the uptake and storage of inorganic carbon by cone-forming cyanobacteria from Yellowstone National Park using high-resolution stable isotope mapping of labeled carbon (H^13^CO_3_^−^) and immunoassays. Observations and incubation experiments in actively photosynthesizing enrichment cultures and field samples reveal the presence of abundant cyanophycin granules in the active growth layer of cones. These ultrastructurally heterogeneous granules rapidly accumulate newly fixed carbon and store 18% of the total particulate labeled carbon after 120 mins of incubation. The intracellular distribution of labeled carbon during the incubation experiment demonstrates an unexpectedly large contribution of PEP carboxylase to carbon fixation, and a large flow of carbon and nitrogen toward cyanophycin in thin filamentous, non-heterocystous cyanobacteria. This pattern does not occur in obvious response to a changing N or C status. Instead, it may suggest an unusual interplay between the regulation of carbon concentration mechanisms and accumulation of photorespiratory products that facilitates uptake of inorganic C and reduces photorespiration in the dense, surface-attached communities of cyanobacteria from Subsection III.

## Introduction

Cyanobacteria fix inorganic carbon using the enzyme ribulose 1,5 bi-phosphate carboxylase oxygenase (RuBisCO) [1] and commonly encapsulate this enzyme in polyhedral inclusions called carboxysomes to improve the carboxylation efficiency [2]–[5]. Cyanobacterial cells can also contain other carbon- and nitrogen-rich inclusions, including cyanophycin, but the roles of these inclusions are less well understood. Cyanophycin, a non-protein, non-ribosomally produced branched polypeptide is composed of an aspartic acid backbone and (L)-arginine side chains [6]–[8] and is commonly found in stationary phase cells [9]–[11]. Some studies suggest that cyanophycin is a dynamic buffer for fixed nitrogen in cyanobacterial heterocysts and unicellular cyanobacteria [11], [12] and in some filamentous, non-heterocystous, nitrogen-fixing cyanobacteria [13]. However, less is known about cyanophycin and other cellular inclusions in non-heterocystous cyanobacteria [14] and filamentous, non-heterocystous benthic cyanobacteria (Subsection III) [15], although the latter are common in various marine and terrestrial benthic environments [16]–[19]. For example, our recent study reported the accumulation of particulate organic carbon in the abundant, 150–750 nm wide, dark intracellular granules within photosynthetically active, thin, cone-forming filamentous cyanobacteria from Yellowstone National Park (YNP) [19]. These cyanobacteria are classified into filamentous, non-heterocystous Subsection III, based on the analysis of 16S rRNA gene sequences and microscopic observations of both field and laboratory samples [19]. The large, dark granules are common in modern coniform mats and visible by light microscopy, so much so that they inspired the name *Phormidium tenue* var. *granuliferum* [20] for cone-forming cyanobacteria. The composition and function of these granules have not been determined to date.

Here, we analyze the composition and ultrastructure of these intracellular inclusions by transmission electron microscopy (TEM) and immunogold labeling. We also track the flow of labeled carbon in cone-forming cyanobacteria by incubation experiments, isotopic labeling and nano-scale secondary ion mass spectrometry (SIMS). These experiments, performed in the laboratory enrichment cultures of cyanobacteria from YNP and in alkaline hot springs in YNP, identify the large inclusions within cyanobacterial cells as cyanophycin and demonstrate ultrastructural and compositional heterogeneity within individual inclusions. Labeling experiments coupled with nano-scale secondary ion mass spectrometry (SIMS) and transmission electron microscopy (TEM) reveal a rapid flow of fixed carbon toward cyanophycin, and the accumulation of a substantial fraction of particulate organic carbon in this reservoir. This surprising pattern, which involves the channeling of carbon toward phosphoenolpyruvate (PEP) carboxylase and the synthesis of cyanophycin, may be an important adaptation of modern, cone-forming cyanobacteria, and other Subsection III cyanobacteria to life in biofilms and chemically stratified environments.

## Results and Discussion

### Detection and Characterization of Cyanophycin Granules

**Figure 1:**
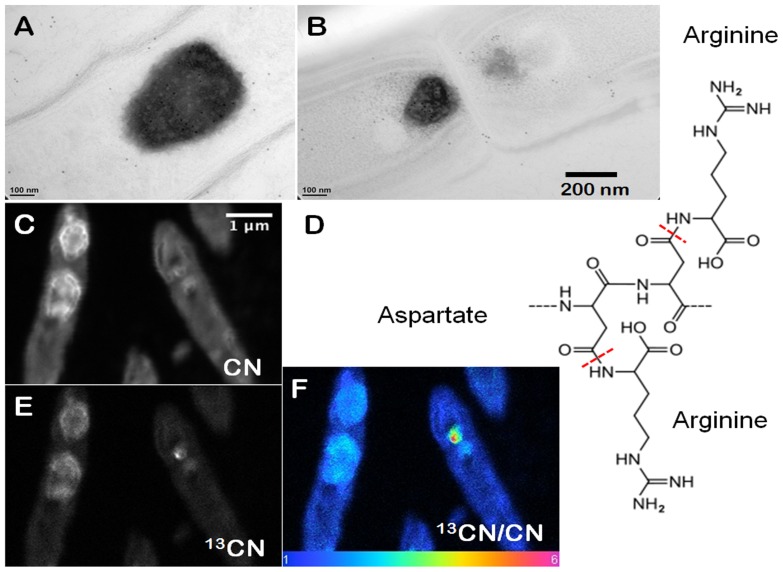
Composition and appearance of cyanophycin granules. (**A**) TEM image of cyanophycin granules within filamentous cyanobacterial cells from field samples (YNP). The sample was immunogold labelled by Rabbit anti-L-Arginine at a dilution of 200X. (**B**) TEM image of filamentous cyanobacterial cells from a lab cone. The sample was immunogold labelled by Rabbit anti-L-Arginine at a dilution of 400X. Scale bar is 200 nm for (**A** and **B**). (**C**) High resolution nano-scale SIMS imaging of CN^-^, showing rimmed globular clusters within cyanophycin granules. (**D**) The suggested chemical structure of cyanophycin, with aspartate as the backbone and arginine as the side chain. (**E, F**) High resolution nano-scale SIMS map of ^13^CN^−^ in the granules of cyanophycin from cyanobacteria grown in the laboratory. (**F**) Ratio map of ^13^CN^−^ to ^12^CN^−^ in cyanophycin granules. Scale bar is 1 µm for (**C, E, F**).

**Figure 2:**
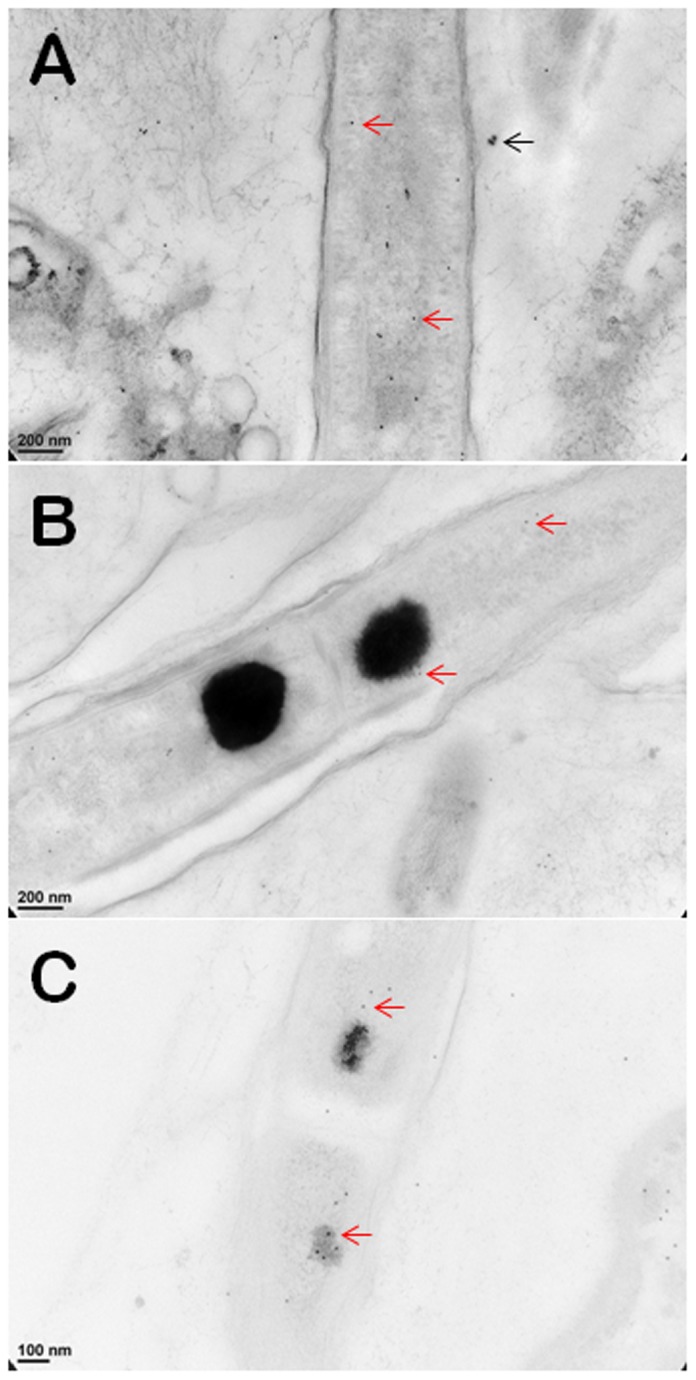
Immunogold TEM images of cyanobacterial cells from YNP samples. All cells were immunogold labelled by Rabbit anti-RuBisCO (large subunit, form I and II, AS03 037) at a dilution of 1000X. The gold particles are uniformly sized and dark, two examples are marked by red arrows in (**A, B, C**). (**A**) The distribution of immunogold particles tagging free RuBisCO in a cell lacking cyanophycin or carboxysomes. SiO_2_ around the cell is marked by a black arrow, these granules are bigger than gold particles. (**B**) The absence of immunogold particles in cyanophycin granules indicates the absence of RuBisCO. (**C**) Immunogold particles tagging RuBisCO in small inclusions suggest the presence of morphologically atypical carboxysomes. These inclusions are rare and are found in the same areas where large cyanophycin granules typically occur. Few gold particles outside the cell in (**A, B, C**) indicate a low level backgroung. Scale bars: 200 nm in (**A, B**), and 100 nm in (**C)**.

Cyanobacteria that form cones and pinnacles in YNP and laboratory cultures contain prominent, 150–750 nm wide, carbon- and nitrogen-rich inclusions that appear dark in TEM images [19]. These granules rapidly concentrate new organic carbon during active photosynthesis and are localized close to the septa that separate cells in filaments (Fig. 1). To test for the presence of RuBisCO in these granules and elsewhere in the cells, we used Rabbit anti-RuBisCO for immunogold labeling. Antibodies for RuBisCO were present primarily in the cytoplasm (Fig. 2A) and much more rarely in the ∼100 nm wide inclusions (Fig. 2C), but were absent from the large granules (Fig. 2B). Instead, immunogold labeling using Rabbit anti-L-arginine demonstrated abundant L-arginine in the large, dark granules (Figs. 1A and B, Fig. S1; see Fig. S2 for images of negative controls). Together, these experiments identified the large, ∼150–750 nm wide, dark granules in thin filamentous cyanobacteria as cyanophycin.

High resolution nano-scale SIMS mapping of the CN^-^ ion species, used to track proteinaceous compounds [21], revealed the ultrastructure of cyanophycin granules at an unprecedented scale. Each large, ∼150–750 nm wide granule contained densely clustered, rimmed, ∼100 nm wide subcompartments with a distinct outer rim (Fig. 1C). The contrast between the interiors and rims of subcompartments stems from a higher intensity of the CN^-^ in the rims, suggesting compositional heterogeneity. A higher intensity of CN^-^ in the rims is consistent with an enrichment of nitrogen-rich L-arginine in the rims and a higher abundance of nitrogen-poor L-aspartate in the interiors of the subcompartments (Fig. 1D). Alternatively, the heterogeneous distribution of CN^-^ within the bright rims and the duller interiors of cyanophycin granules, respectively, may track cyanophycin and another peptide-rich compound, respectively.

### Carbon Accumulation and Storage in Cyanophycin

**Figure 3:**
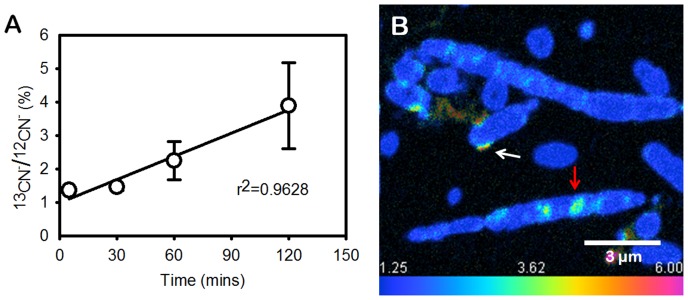
Temporal dynamics of ^13^CN^−^ in cyanophycin. (**A**) Average accumulation of labeled carbon within the cells (N>10) at different time points. (**B**) Accumulation of labeled carbon within cyanophycin after 30 min of incubation. The ^13^CN^−/12^CN^−^ ratio was 2.5% (i.e., twice above the 1.25% background). Cyanophycin is marked by a red arrow. Labeled carbon is also present in or close to cell envelopes (white arrow). Scale bar is 3 µm.

**Figure 4:**
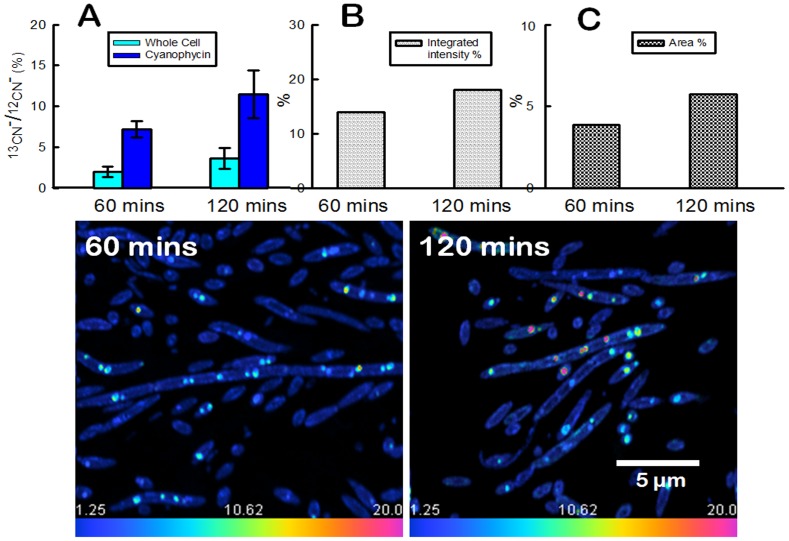
Carbon accumulation and storage within cyanophycin over time. (**A**) Isotopic enrichment in cyanophycin granules and cell cytosol after 60 and 120 mins of incubation (see images “**60 mins**” and “**120 mins**”). (**B**) Labeled carbon in cyanophycin granules expressed as the percentage of total labeled carbon in samples shown in (**60 mins, 120 mins**). (**C**) Percentage of cellular area that is occupied by cyanophycin granules in (**60 mins, 120 mins**). (**60 mins**) Nano-scale SIMS image of large cyanophycin granules in filamentous cyanobacteria ∼120 µm below the surface of the cone after 60 min of incubation in the labeling medium. (**120 mins**) Nano-scale SIMS image of cyanophycin granules in filamentous cyanobacteria ∼120 µm below the surface of the cone after 120 mins of incubation in the labeling medium. Cyanophycin granules in (II) contain more labeled carbon and are larger. Scale bar is 5 µm for (**D**, **E**).

**Figure 5:**
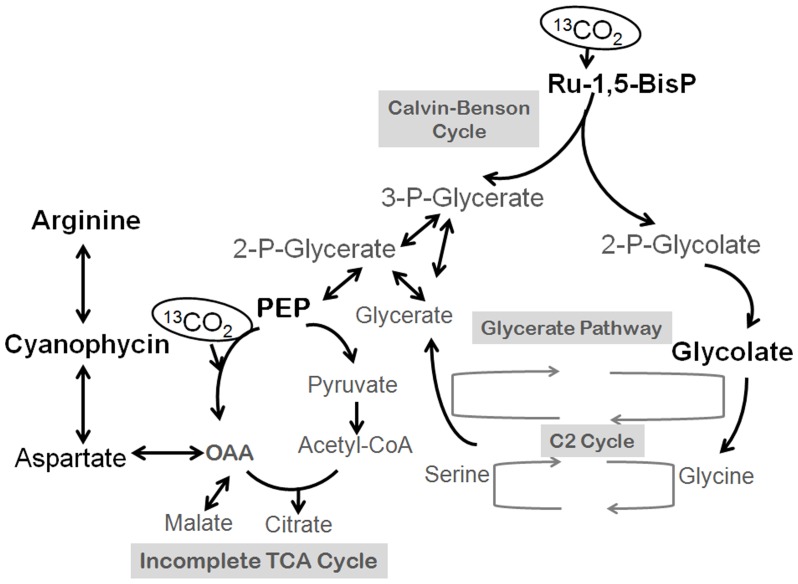
Proposed scheme of central carbon and nitrogen metabolism in cone-forming cyanobacteria. Adapted from Huege et(RuBisCO) detected in this study are marked in dark.

Studies of carbon flux through cone-forming communities in YNP and laboratory enrichment cultures suggested a central role for cyanophycin granules in the accumulation and storage of fixed carbon in the active growth layer of cones (Fig. 1 E, F) [19], [22]. To track the flow of newly fixed carbon through cyanobacterial cells in time, we grew coniform mats in the laboratory, in media containing a high concentration of inorganic carbon (C_i_). Small cones were cut out, and incubated in a medium that contained ^13^C-labeled bicarbonate in the presence of a light source (Methods). Cones labeled in this manner were sampled at 5, 30, 60 and 120 mins after the start of the incubation. High resolution nano-scale SIMS mapping of ^12^CN^−^ ions and ^13^CN^−^ ions (see Methods) of thin sections through cones showed an increasing accumulation of labeled particulate carbon in time (Fig. 3A). After 5 minutes of incubation, labeled carbon was only detectable in the envelopes of filamentous cyanobacteria (Fig. S3). After 30 minutes, labeled carbon was present primarily in the cyanophycin granules (Fig. 3B), where the ^13^CN^−/12^CN^−^ ratio was twice higher than the background (1.25%, in resin). After an additional 30 minutes (60 min total), cyanophycin granules occupied 3.9% of the cell area and accounted for 13.9% of the particulate labeled carbon. The isotopic enrichment, expressed as ^13^CN^−/12^CN^−^ ratio, was 2.0% (±0.6%) and 7.2% (±1.0%) in the cytosol and cyanophycin, respectively. After 120 mins, the large cyanophycin granules were enriched isotopically by 11.5% (±2.9%) relative to the 3.6% (±1.3%) enrichment of the cytosol (Fig. 4A). At this time, cyanophycin granules accounted for about 5.7% of the cell area and for 18% of the labeled carbon in cyanobacterial cells (Fig. 4B, C). The inflow of ^13^C into cyanophycin requires that cone-forming cyanobacteria rapidly channel fixed carbon via phosphoenolpyruvate (PEP) carboxylase to PEP pool and aspartate (Fig. 5). These fluxes of fixed carbon resemble the reported rapid production of labeled aspartate and PEP in the unicellular cyanobacterium *Synechocystis* sp. strain PCC 6803 acclimated to low C_i_ and subject to a pulse of ^13^C-labeled medium with very high C_i_ [23]. Longer term labeling experiments, pulse-chase experiments, and the tracking of soluble metabolites could evaluate fluxes of carbon through different pools of carbon in filamentous, cone-forming cyanobacteria, and in other cells within the community.

Photosynthetically active cone-forming cyanobacteria from untreated YNP samples and laboratory cultures contain RuBisCO (Fig. 2) and rare carboxysomes [19]. These organisms also excrete glycolate in the presence of high C_i_ under our laboratory conditions [24]. Therefore, cone-forming cyanobacteria use RuBisCO to fix inorganic carbon under all tested experimental and field conditions (Fig. 4). Surprisingly, the intracellular distribution of labeled particulate organic carbon in cone-forming cyanobacteria also provides evidence for a substantial flow of fixed carbon toward PEP carboxylase, and the synthesis of aspartate, arginine and cyanophycin (Fig. 5). This occurs both in laboratory enrichment cultures (Figs. 1, 4) and in field cones from YNP [19]. PEP carboxylase contributes to carbon fixation in the stationary cultures of unicellular cyanobacteria *Aphanocapsa* 6308 [25] and in the cultures of *Synechocystis* sp. PCC 6803 acclimated to low concentration of inorganic carbon (∼ 60 µM C_i_) but experiencing a pulse of high C_i_ [23]. In contrast, we speculate that cyanobacterial filaments in modern coniform mats channel fixed carbon toward PEP and synthesize cyanophycin even when growing actively and in the presence of both low and high C_i_. We infer this from the following: 1) cyanophycin granules are common in the actively growing tips of laboratory and field cones even before the transfer to the labeling medium [19], [22] (Methods); 2) cyanobacteria in the tips of laboratory cones grow in solutions containing ∼ 25 mM C_i_, i.e., two order of magnitude higher concentration than low C_i_ conditions in *Synechocystis* cultures (0.18 mM); and 3) cyanobacterial cones were transferred to labeling solutions with ∼ 25 mM C_i_ (Methods).

Studies of unicellular cyanobacteria that do not fix nitrogen and studies of N- and C-fixing cyanobacteria show that cyanophycin is typically synthesized by cyanobacteria that transition between a nitrogen-replete to a nitrogen-poor regime, and may be degraded during the transition from low to high C_i_ concentrations to balance the internal C/N ratio [11]–[13], [23], [26], [27]. The observed flux of carbon toward PEP carboxylase and cyanophycin under our laboratory conditions did not occur in response to a changing N regime because both the growth and the incubation media contained comparable concentrations of C_i_ and N_i_ (∼25 mM DIC and 1.8 mM nitrate). Cells grown and incubated under these conditions contained rare carboxysomes and abundant cyanophycin granules. Cyanophycin is also synthesized during typical growth conditions in coniform mats in YNP, because cyanobacteria in the field cones contain abundant cyanophycin granules and rare carboxysomes [19]. The field conditions are likely characterized by less than 5 mM DIC and 0.05 mM N_i_ in the solution above the coniform mats [28], [29]. Because the field structures grew acclimated to low N_i_, but were labeled in the medium that contained 1.8 mM nitrate [19], it is possible that our ^13^C-labeling experiment in the field probed the response to a changing N regime. Notably, we observed similar patterns of C flow through cyanobacterial cells both in the laboratory and in the field [19].

Actively growing cone-forming cyanobacteria from cone tips do not appear to synthesize cyanophycin in response to a changing N regime. Instead, morphological, chemical and isotopic patterns observed in the macroscopic, surface-attached, diffusion-limited [22], [30] or even carbon-limited cones [28] suggest that cone-forming cyanobacteria may synthesize cyanophycin in response to the low or changing C/O ratios. Mutants of *Synechococcus* sp. strain PCC 6803 that lack carboxysomes and are impaired in the ability to concentrate carbon and those that accumulate the products of RuBisCO oxygenation such as glycolate (Fig. 5) channel larger portions of fixed carbon toward PEP carboxylase [23], [31]. Wild-type cone-forming cyanobacteria exhibit similar trends under our experimental conditions (∼25 mM C_i_ in the bulk medium): they excrete glycolate, and incorporate aspartate derived from the PEP carboxylase pathway into cyanophycin granules (Fig. 5). Given that phosphorespiratory products, including glycolate, inhibit enzymes of the Calvin Benson Basham pathway [32], [33], and that the clumping behavior of cone-forming cyanobacteria promotes the removal of glycolate and does not occur at low oxygen concentrations [24], both clumping and the increased flux of carbon through PEP carboxylase may compensate for some of the inhibitory effects of photorespiration. This pathway may be of particular importance in dense, surface-attached cyanobacterial communities where the transport of metabolites is limited by diffusion [22]. These hypotheses can be explored by quantifying the production of glycolate and cyanophycin in coniform mats and dispersed mats exposed to different C/O ratios and by further exploring the dynamics of carbon cycling through cyanophycin granules.

Cyanophycin seems to play an extraordinary role in the cycling of carbon in thin filamentous cyanobacteria from modern cone-forming aggregates. The previously unrecognized flux of fixed carbon toward PEP carboxylase and the incorporation of this carbon into cyanophycin directly links the cycles of carbon, nitrogen and oxygen, and may be one of many unusual adaptations of thin, filamentous cyanobacteria to life in macroscopic aggregates in the presence of low flow [19], [20], [22], [24], [30], [34]. Cyanobacteria from Subsection III that live in environments other than hot springs also have the capacity to synthesize cyanophycin and often do so [16]–[18], [35], under conditions that remain to be elucidated.

## Materials and Methods

### Culturing and Isotope Labeling

Field work was performed in August 2008 and 2009 in Mound Spring at Sentinel Meadows, Yellowstone National Park (YNP) under the permit YELL-2008-SCI-5758 and coniform mats were sampled and transported as described previously [19], [34]. Laboratory enrichment cultures were grown as described previously [36], in 200 ml glass beakers at 45°C, in a modified Castenholz D medium, on silica or aragonite sand as solid substrate. Genomic DNA was extracted from cones grown in laboratory enrichment cultures. Primer sets of 27F/1492R for universal bacterial 16s rRNA sequences, and cyanobacteria-specific primers 338F/781R, 359F/781R were used for PCR and nested PCR as described in references [19], [34]. The cultures were grown at a light intensity of 180 µE/m^2^/s using cold fluorescent light with a day-night cycle of 12–12 hours. Three weeks after the inoculation, and 3–7 days after the appearance of ∼ 5 mm tall and ∼ 2 mm wide cyanobacterial cones in laboratory enrichment cultures, the liquid medium above the surface-attached cyanobacteria was replaced by the fresh, modified Castenholz D medium [36], [37] that contained 1.8 mM NO_3_^−^ and ∼25 mM sodium bicarbonate enriched in ^13^C (98 atom%, Isotec, Miamisburg, Ohio) and equilibrated with 5% ^13^CO_2_ in the culture headspace. Cones were incubated for 5, 30, 60 and 120 mins in separate containers.

### Isotopic Mapping of Labeled Carbon by Nano-scale SIMS

After the incubation with isotopically labeled inorganic carbon, the samples were fixed with 2.5% glutaraldehyde in 0.1 M sodium cacodylate buffer (pH 7.4), washed with the same buffer, and post-fixed in 1% osmium tetroxide and 1.5% potassium ferrocyanide. The fixed samples were dehydrated in a gradient series (30–100%) of acetone/water solutions and embedded in epoxy resin (Epon). Thin sections (300 nm) were obtained using a Reichert-Jung Ultracut E ultramicrotome with a diamond knife. The sections were collected and transferred onto a clean Si chip for SIMS imaging.

Nano-scale Secondary Ion Spectrometry (Nano-scale SIMS) analyses were carried out at the Institut Curie, Orsay, France using a NanoSIMS-50 ion microprobe (Cameca, Gennevillieres, France). Measurement conditions were described in details in previous studies [19], [22]. The optical system in the microprobe was adapted to ensure high transmission with a high mass resolution. The primary beam was operated in the probe scanning mode over the sample surface to create elemental and isotopic images. To obtain high resolution images of individual cell details, the raster was reduced to 6, 12 and 20 µm, with the probe size smaller than 100 nm and an intensity of about 1 pA. Images were created for ion species ^12^C^−^,^ 13^C^−^, ^12^C^14^N^−^, ^13^C^14^N^−^ and ^32^S^−^. The dwell time per pixel was 30 ms for high resolution images. High mass resolution setup was used to separate ^13^C^14^N^−^ from the isobaric ^12^C^15^N^−^ species on one hand [21], and on the other hand, to prevent the mass interference from ^11^B^16^O^−^ species that was likely present in the embedding resin.

### Nano-scale SIMS Data Processing

Ion species ^12^C^14^N^−^ and ^13^C^14^N^−^ were used to detect carbon associated with biomass, as they have higher intensity and are more statistically reliable than ^12^C^−^ and^ 13^C^−^. The ratio (%) of ^13^C^14^N^−/12^C^14^N^−^ was used to quantify the uptake of carbon (enrichment over the background). Color ratio image was generated by using OpenMIMS, an ImageJ plugin [38] developed by Claude Lechene’s Laboratory. OpenMIMS allows the visualization of ratio value in a HSI (Hue-Saturation-Intensity) image [39], [40]. To compute the average ^13^C^14^N^−/12^C^14^N^−^ ratio in the cells in each image, the information pertaining to microbial biomass (enriched in ^13^C) was separated from the background resin (natural abundance of ^13^C) by a mask. The binary mask was generated by adjusting the threshold level on the sum image of ^13^CN^−^ and ^12^CN^−^. Each pixel containing biomass was assigned a value of 1, other pixels were assigned a value of 0. A ^13^CN^−/12^CN^−^ ratio map was obtained by multiplying the measured ^13^CN^−/12^CN^−^ ratio image with the binary mask image to eliminate the resin background. The ratio characteristic for the unlabeled controls (N = 3, numerical value 1.25) was first subtracted from each pixel value, and pixel values in the corrected ratio of ^13^CN^−/12^CN^−^ were integrated over all pixels. The isotopic enrichment in carbon concentrating granules was determined using ultra-high resolution images.

### Immunohistochemistry and Transmission Electron Microscopy Imaging

Samples of cyanobacterial cones were frozen using a high pressure freezer (Leica EMPACT2) at pressure 2000–2050 bar. Freeze substitution was performed in anhydrous ethanol (containing 0.2% uranyl acetate and 0.2% glutaraldehyde) using a Leica EM AFS2 (automatic freeze substitution). The samples were kept at −90°C for 3 days, at −60°C for 1 day, at −20°C for 1 day, at 0°C for 1 day and were then warmed to room temperature. The LR Gold resin was used for infiltration and embedding. Ultrathin sections (90–120 nm) were obtained using a Reichert Ultracut S or Leica EM UC6ultramicrotome (Leica, Vienna, Austria) with a diamond knife. The sections were collected and transferred onto 100-mesh nickel grids.

In immunogold labeling experiments, the individual grids were floated on Tris-buffered saline (TBS) for 15 mins, and 1% bovine serum albumen (BSA) in TBS for 15 mins. To determine whether L-Arginine was present in the large intracellular granules, the grids were incubated with Rabbit anti-L-Arginine (Acris, AP31587SU-S) diluted 200X, 400X and 800X in TBS and 1% BSA for 1 hour (Fig. S1). Normal Rabbit Immunoglobulin G (IgG) (Millipore, 12–370), diluted 200X, 500X and 800X, was used as a control (Fig. S2). After 4 washes with TBS, the grids were floated on an excess (1∶20 dilution) of 12 or 18 nm colloidal Donkey anti-rabbit IgG (Jackson Immuno Research, West Grove, PA, USA) at room temperature for 1 hour. The grids were washed sequentially with 3 drops of TBS, followed by three washes with ddH_2_O. After immunogold labeling, the sections were stained with 5% uranyl acetate in water for 10 mins and 0.4% lead citrate in water for 6 mins. Sections were imaged using a Philips CM 100 Transmission Electron Microscope at 80 kV and recorded by a GatanOrius CCD camera at the Plant Cell Biology Core Lab, Institute of Plant and Microbial Biology, Academia Sinica. To determine the distribution of RuBisCO in thin filamentous cyanobacteria, immunogold labeling was also performed using Rabbit anti-RuBisCO large subunit (1000 dilution in TBS and 1% BSA, Agrisera, AS03 037) following the protocol described above (Fig. 2).

## Supporting Information

Figure S1 (**A, B**) TEM image of cyanophycin granules within filamentous cyanobacterial cells from YNP cones. The samples were immunogold labelled by Rabbit anti-L-Arginine at 200X and 400X dilutions. (**C**) TEM image of cyanobacteria from a lab culture. The samples were immunogold labelled by Rabbit anti-L-Arginine at a dilution of 800X. Scale bar for (**A, B, C**) is 100 nm. Small amount of L-Arginine may be present in the cell, accounting for a certain level of background.
(TIF)

Figure S2 TEM image of controls for immunogold labelling using Normal Rabbit IgG antibody (Millipore, 12–370). (**A**, **B**, and **C**) show samples that used the antibody at dilutions of 200X, 500X and 800X, respectively.(**A** and **C**) are field samples from YNP, **B** shows cells from a laboratory culture. The 200X dilution generates a low background cross-reaction, 500X and greater dilutions show zero cross-reaction. The scale bars are 0.5 µm for (**A** and **C**), and 0.2 µm for (**B**).
(TIF)

Figure S3 Nano-scale SIMS isotopic ratio map of ^13^CN^-^ to ^12^CN^−^ for filamentous cyanobacteria from the tip of a cone after a 5 min incubation. Labeled carbon scattered around cell envelopes, suggesting that the initial carbon incorporation possibly occurs there. Scale bar is 5 µm.
(TIF)
