# Supplementary figures and images for: Cyanophycin Mediates the Accumulation and Storage of Fixed Carbon in Non-Heterocystous Filamentous Cyanobacteria from Coniform Mats
Source: PLoS One. 2014 Feb 7;9(2):e88142. doi: 10.1371/journal.pone.0088142 (PMC3917874; doi:10.1371/journal.pone.0088142)

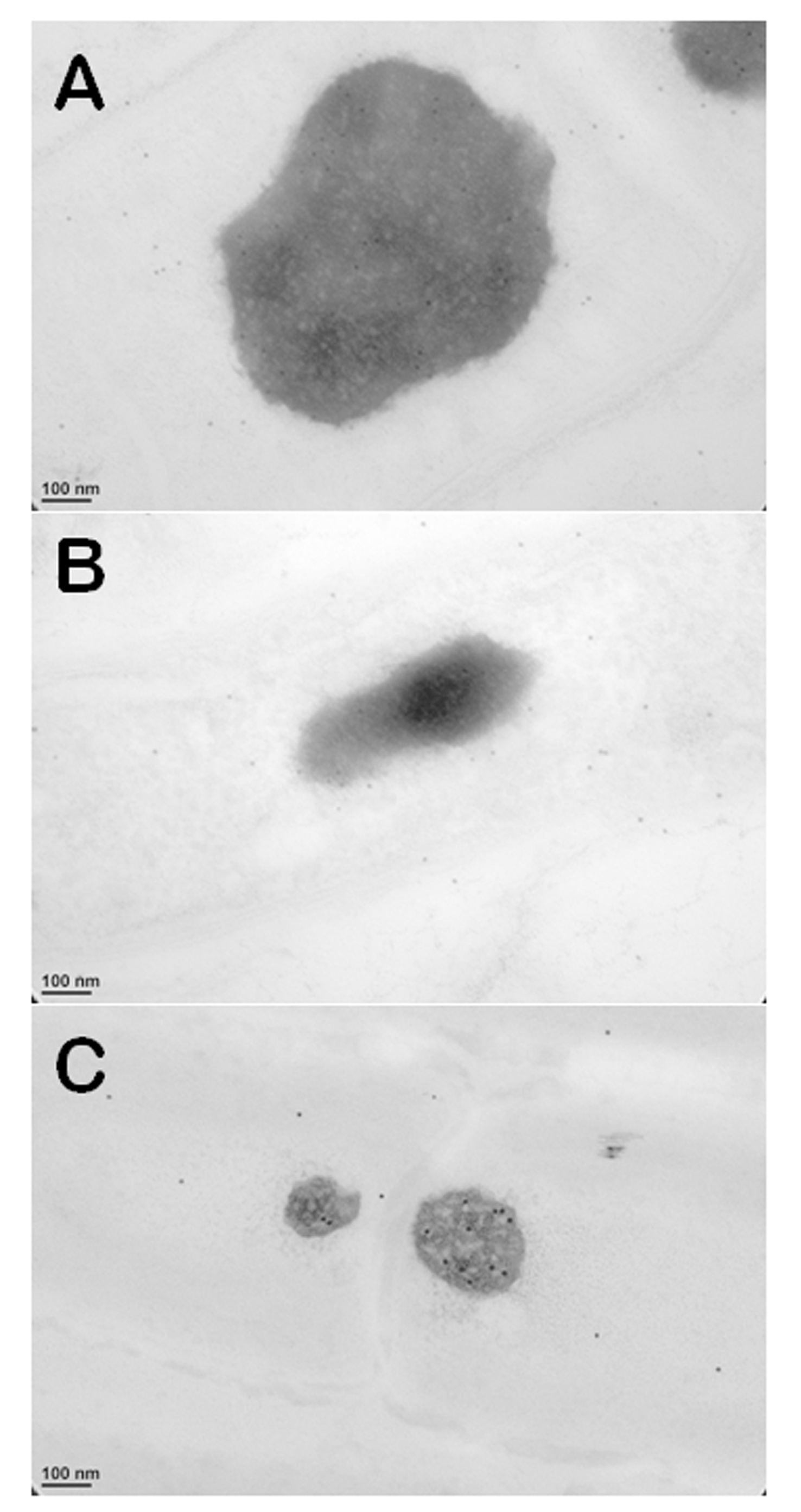

Supplement: Figure S1 — (A, B) TEM image of cyanophycin granules within filamentous cyanobacterial cells from YNP cones. The samples were immunogold labelled by Rabbit anti-L-Arginine at 200X and 400X dilutions. (C) TEM image of cyanobacteria from a lab culture. The samples were immunogold labelled by Rabbit anti-L-Arginine at a dilution of 800X. Scale bar for (A, B, C) is 100 nm. Small amount of L-Arginine may be present in the cell, accounting for a certain level of background. (TIF) [file pone.0088142.s001.tif]

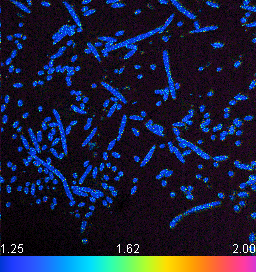

Supplement: Figure S3 — Nano-scale SIMS isotopic ratio map of 13CN- to 12CN− for filamentous cyanobacteria from the tip of a cone after a 5 min incubation. Labeled carbon scattered around cell envelopes, suggesting that the initial carbon incorporation possibly occurs there. Scale bar is 5 µm. (TIF) [file pone.0088142.s003.tif]
